# Supplementary material for: Alterations of Extracellular Matrix Mechanical Properties Contribute to Age-Related Functional Impairment of Human Skeletal Muscles
Source: Int J Mol Sci. 2020 Jun 2;21(11):3992. doi: 10.3390/ijms21113992 (PMC7312402; doi:10.3390/ijms21113992)
Supplement: Supplementary file 1 [file ijms-21-03992-s001.zip › IJMS-803657 supplementary for conversion.docx]

Supplementary materials

Constitutive models

In a previous work [1] the uni-axial passive behaviour of the fibres was described in terms of nominal stress as:

| (s1) |
| --- |

where the scalar *A* is a stress-like parameter and is the stretch, deduced from the deformed length of the sarcomere *L* and its initial length *L*0. The passive behaviour of the bundle in uni-axial tensile conditions has been modelled as:

| (s2) |  |
| --- | --- |

with the definition , where *L* is the current length of the bundle and *L*0*b* its stress-free length. The scalar is the area fraction of ECM and the stress-like parameter is related to its stiffness; in particular, it can be demonstrated that the initial (i.e. at small strains) longitudinal elastic modulus is given by .

We have performed a multiple fitting equation (s1) on the whole set of data of the fibres for an initial length of 2.44 μm, finding the value 9.96 kPa for the parameter *A*. With this data we have performed a separate fitting of equation (s2) on the data of young bundles (with ) and elderly bundles (with ) for the same value , obtaining for elderly bundles and for young bundles.

Supplementary Figure S1


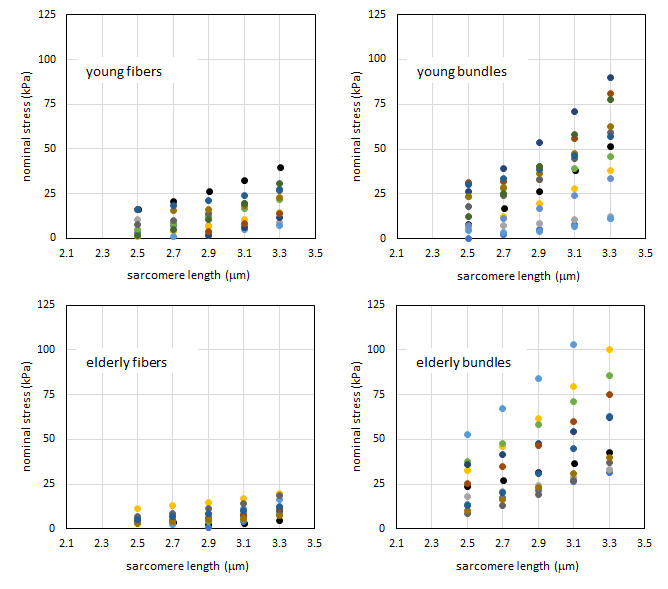


**Figure S1.** Individual data obtained after interpolation of experimental data from each tensile test on fibers and bundles. The graphs are proposed with the same scale for the stress, to show the different response among the four groups considered. Each fiber or each bundle is identified by a different color. Young samples: 12 fibers and 13 bundles. Elderly samples: 11 fibers and 11 bundles.

Supplementary Table 1

A) subjects (all males) who volunteered for the biopsy sampling

| **Young Group** | **Age (years)** | **Body mass (kg)** | **Height (cm)** | **BMI** | **Physical status** |
| --- | --- | --- | --- | --- | --- |
| 1 | 21 | 77.2 | 188 | 21.8 | Mod. active |
| 2 | 21 | 105.4 | 192 | 28.5 | Mod. active |
| 3 | 21 | 69.9 | 182 | 21.2 | Mod. active |
| 4 | 19 | 67.5 | 178 | 21.3 | Mod. active |
| 5 | 21 | 75.5 | 181 | 23.0 | Mod. active |
| mean ±SE | 20.6 ± 0.4 | 79.1 ± 6.81 | 184.2 ± 2.53 | 23.16 ± 1.37 |  |
| **Elder Group** | **Age (years)** | **Body mass (kg)** | **Height (cm)** | **BMI** | **Physical status** |
| 1 | 69 | 78.7 | 170 | 27.2 | Mod. active |
| 2 | 65 | 85 | 171 | 29.1 | Mod. active |
| mean ±SE | 67 ± 2 | 81.85 ± 3.1 | 170.5 ± 0.5 | 28.15 ± 0.91 |  |

B) subjects who gave their consent to use for research a sample collected during surgery

| **Subject** | **Gender** | **Age** |
| --- | --- | --- |
| 1 | F | 55 |
| 2 | M | 42 |
| 3 | M | 41 |
| 4 | M | 27 |
| 5 | M | 39 |
| 6 | M | 18 |
| 7 | F | 49 |
| 8 | M | 45 |
| 9 | M | 32 |
| 10 | M | 63 |
| 11 | M | 79 |
| 12 | M | 79 |
| 13 | F | 94 |
| 14 | M | 94 |
| 15 | M | 61 |
| 16 | M | 74 |

**References**

1. Marcucci, L.; Bondì, M.; Randazzo, G.; Reggiani, C.; Natali, A.N.; Pavan, P.G. Fibre and extracellular matrix contributions to passive forces in human skeletal muscles: An experimental based constitutive law for numerical modelling of the passive element in the classical Hill-type three element model. *PLoS ONE* **2019**, *14*, doi:10.1371/journal.pone.0224232.

| 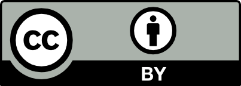 | © 2020 by the authors. Submitted for possible open access publication under the terms and conditions of the Creative Commons Attribution (CC BY) license (http://creativecommons.org/licenses/by/4.0/). |
| --- | --- |
